# Sterilisation by "Frying"

**Published:** 1898-01-15

**Authors:** 


### STERILISATION BY "FRYING."

There can be no doubt that the most perfect method of sterilisation, where it can be applied, is by heat. Baking, however, is a more or less uncertain process, while boiling is destructive to many substances. Moreover, the boiling temperature is so little above that which is fatal to microbic life that a considerable length of exposure to such a temperature is necessary, if one is to be sure that the process has been effectually carried out. Frying, however, is another matter. Olive oil at a temperature of 160 to 180 deg. Centigrade acts very quickly, and with great power. Professor Wright, of Netley, says that to obtain complete sterilisation of an instrument it suffices to dip it for an instant into the hot oil, and that in the case of syringes it is sufficient to fill them twice with oil at the temperature mentioned.

The temperature of the heated oil may be determined by a thermometer, but it is often more convenient to adopt the rough-and-ready methods of the cook by the aid of a bit of bread-crumb. "It will be found that the bread-crumb will become *brown* and crisp as soon as a temperature of 160 to 180 is reached." For the sterilisation of syringes all that is necessary is to heat a little oil in a spoon over a spirit lamp, testing it from time to time by bits of bread-crumb, and, when the proper temperature has been attained, to fill the syringe twice with hot oil. All microbial infection will then have been destroyed.

